# Editorial: Distinct phenotype but same genotype: hints for the diversity of phenotypes in ciliopathies

**DOI:** 10.3389/fmolb.2025.1753098

**Published:** 2025-12-11

**Authors:** John A. Sayer, Heymut Omran, Ewa Zietkiewicz

**Affiliations:** 1 Biosciences Institute, Faculty of Medical Sciences, Newcastle University, Central Parkway, Newcastle upon Tyne, United Kingdom; 2 Renal Services, Newcastle Upon Tyne Hospitals NHS Foundation Trust, Newcastle upon Tyne, United Kingdom; 3 NIHR Newcastle Biomedical Research Centre, Newcastle upon Tyne, United Kingdom; 4 Department of Pediatrics, University Hospital Muenster, Muenster, Germany; 5 Institute of Human Genetics Polish Academy of Sciences, Poznan, Poland

**Keywords:** genetic diagnoses, primary cilary dyskinesia, personalisable medicine, DNAH5, *ex vivo*

Primary ciliary dyskinesia (PCD) represents one of the most complex inherited disorders of mucociliary dysfunction, characterised by marked clinical heterogeneity and a challenging diagnostic pathway (Shoemark et al., 2025). Mutations in more than 60 different genes can result in abnormal ciliary clearance of the airway system explaining genetic heterogeneity as well phenotypic variability. This Research Topic highlights a timely and compelling overview of the current state of PCD diagnostics, revealing both the progress made and the significant gaps that should be addressed if patients are to receive timely, accurate, and mechanism-informed care.

Two studies investigated the clinical phenotype in genetically confirmed PCD individuals (Schramm et al.; Rizk et al.). Both studies show that normal ultrastructure PCD can be best diagnosed by genetic testing. In addition, Schramm et al. examine the performance of PICADAR, in regards to the sensitivity of one of the most widely used clinical prediction tools in PCD (Schramm et al.). Interestingly, this was the first study, which included genetically confirmed PCD cases. Previous studies focussed mostly on abnormal ultrastructure PCD. Although endorsed by the European Respiratory Society, PICADAR’s sensitivity was only 75% in individuals with genetically confirmed disease. Crucially, the tool failed most prominently in two groups of particular clinical relevance: individuals with normal organ laterality and those lacking hallmark ultrastructural defects of motile cilia. In these patients, sensitivity fell to 61% and 59%, respectively. These findings remind us that patients without laterality defects represent a large and diagnostically vulnerable subset of the PCD population. A tool that performs well only when classical features are present poses the risk of delayed or missing diagnoses in less defined cases. Collectively, the presented papers underscore the need for prediction models which would reflect the full phenotypic spectrum of PCD, including presentations that lack “typical” features, on which legacy diagnostic tools were based. Development of novel tools to predict likelihood of PCD are mandatory and should be assessed in large representative PCD populations using international collaborative initiatives such as the ERN LUNG PCD registry (Raidt et al., 2024).

The high allelic heterogeneity underlying PCD is illuminated by an insightful study (De Ceuninck Van Capelle et al.), which probes how distinct mutations within a single gene, *DNAH5*, translate into variation in protein composition and ultrastructure of cilia. Using mass spectrometry and cryo-electron tomography, the authors demonstrate mutation-specific differences in axonemal organisation, including the absence of several newly identified proteins. Both technologies have emerged as novel technologies to enhance our understanding of the molecular pathology in PCD aiding future precision medicine therapeutic approaches.

A report on Kartagener syndrome (Zhang et al.) emphasises how laterality defects remain an important diagnostic clue. It also highlights the continuing discovery of novel *DNAH5* mutations. The paper reinforces two themes: first, the importance of detailed phenotyping including situs anomalies, chronic sinus disease, otitis media, and bronchiectasis, which remain recognisable, albeit not specific, clinical hallmarks; and second, the ongoing expansion of the genetic landscape. Even in one of the best-studied PCD genes, rare and previously unreported variants continue to emerge. Such findings point to the value of integrating careful phenotyping, detailed imaging, and comprehensive genetic analysis.

Another paper (Kalyoncu et al.) illustrates the growing importance of immunofluorescence analysis of ciliary protein composition, particularly in cases where genetics alone is inconclusive. The detection of abnormal protein localisation (such as misdistribution of DNAH5 or GAS8), combined with video microscopy observation of ciliary beating, and nasal nitric oxide measurements, offers a powerful means of resolving equivocal cases. Notably, the identification of a ciliogenesis defect due to *CCNO* deficiency, and the demonstration of DNAH5 protein absence even in heterozygous variant carriers, highlight the power of protein-level interrogation. More broadly, the study demonstrates how international collaboration, particularly in rare diseases, can transform diagnostic yield and push the field beyond gene panels and traditional assays.

All papers elegantly illustrate why one-dimensional diagnostic tests can fall short: PCD is not a uniform entity, but a family of molecularly distinct disorders. Diagnostic tools and future therapies alike must keep pace with this complexity (Table 1). Taken together, these studies merge into a cohesive narrative indicating that PCD diagnostics is evolving, but current tools still fail to capture the full biological and clinical breadth of the disease. Whether through insufficiently sensitive screening tools, gene-level heterogeneity, novel molecular findings, or challenging “borderline” cases that require multidisciplinary expertise, the limitations are clear. Yet these studies also reveal paths forward. Integrating advanced cell/organelle imaging, molecular assays, broader genetic strategies, and international partnerships promises to refine diagnosis, shorten diagnostic journeys, and ultimately support precision-medicine approaches.

**TABLE 1:** Challenges and future opportunities in primary ciliary dyskinesia (PCD) diagnosis.

Challenge in PCD Diagnosis	Description/Underlying Issue	Future Solutions/Opportunities
Limited sensitivity of clinical prediction tools (PICADAR)	Underperforms in patients without laterality defects or hallmark ultrastructural cilia abnormalities	Develop next-generation multimodal prediction models
High phenotypic heterogeneity	Many patients lack classical features, reducing clinical suspicion	Improve clinician education; reanalyse phenotype-diverse datasets
Absence of a single diagnostic gold standard	Diagnosis requires multiple tests, each with limitations	Integrated algorithms combining structural, functional, molecular and genetic data
Genetic heterogeneity and novel variants	Rare variants and >50 implicated genes complicate interpretation	Use whole-genome sequencing; expand variant databases
Mutation-specific molecular differences	Different mutations in the same gene cause distinct axonemal changes	Use advanced molecular tools; develop biomarkers
Limited access to specialised diagnostic tests	If, HSVM, cryo-ET not widely available; expertise varies	International networks; telepathology; training programmes
Inconclusive or borderline genetic/IF findings	Genetic variants of unknown significance or ambiguous protein localisation patterns complicate diagnosis	Standardise genetic and IF indicators; multidisciplinary boards; extended testing
Under-recognised ciliogenesis defects	Disorders due to defects such as *CCNO* deficiency may be overlooked	Increase awareness and IF use in atypical cases
Limited global population data	Genetic and phenotypic data skewed toward certain regions	Expand global registries; integrate multinational datasets; define population-specific profiles of pathogenic variants
Fragmented diagnostic pathways	Patients experience long, multi-step diagnostic journeys	Develop multidisciplinary PCD centres and referral pathways

HSVM, high speed video microscopy; cryo-ET, cryo-electron tomography; IF, immunofluorescence

As the field advances, we must move beyond a narrow conception of PCD defined by classical features, embracing instead a more nuanced understanding grounded in molecular diversity, variable phenotypes, and multidimensional diagnostic frameworks. The work presented here takes important steps in that direction and points to the critical need for next-generation predictive tools that match the complexity of the disorder they aim to detect.
